# A Penalized Mixture Model Approach in Genotype/Phenotype Association Analysis for Quantitative Phenotypes

**DOI:** 10.4137/cin.s3493

**Published:** 2010-04-27

**Authors:** Lang Li, Silvana Borges, Robarge D. Jason, Changyu Shen, Zeruesenay Desta, David Flockhart

**Affiliations:** 1 Division of Biostatistics, Department of Medicine, School of Medicine, Indiana University, Indianapolis, IN; 2 Division of Clinical Pharmacology, Department of Medicine, School of Medicine, Indiana University, Indianapolis, IN. Email: lali@iupui.edu

**Keywords:** genotype/phenotype association, mixture model, pharmacogenetics

## Abstract

A mixture normal model has been developed to partition genotypes in predicting quantitative phenotypes. Its estimation and inference are performed through an EM algorithm. This approach can conduct simultaneous genotype clustering and hypothesis testing. It is a valuable method for predicting the distribution of quantitative phenotypes among multi-locus genotypes across genes or within a gene. This mixture model’s performance is evaluated in data analyses for two pharmacogenetics studies. In one example, thirty five CYP2D6 genotypes were partitioned into three groups to predict pharmacokinetics of a breast cancer drug, Tamoxifen, a CYP2D6 substrate (p-value = 0.04). In a second example, seventeen CYP2B6 genotypes were categorized into three clusters to predict CYP2B6 protein expression (p-value = 0.002). The biological validities of both partitions are examined using established function of CYP2D6 and CYP2B6 alleles. In both examples, we observed genotypes clustered in the same group to have high functional similarities. The power and recovery rate of the true partition for the mixture model approach are investigated in statistical simulation studies, where it outperforms another published method.

## Introduction

Genetic association studies have been widely used to identify risk factors for complex diseases or to predict drug-treatment outcomes (efficacy or toxicity). One important approach is called candidate gene approaches.^1^ It is frequently selected to investigate genes in known signaling and metabolic pathways. This approach typically narrows gene targets to a handful of candidates deemed to have a stronger potential of affecting outcomes. Consequently, it is feasible to investigate a dense SNP set per gene. For example, in our pharmacokinetics study of Tamoxifen in breast cancer patients, 35 CYP2D6 alleles were investigated from more than 70 known CYP2D6 polymorphisms.

In a candidate gene study, multiple SNPs per gene usually lead to many haplotypes or alleles, creating high genotype dimensions for genotype/phenotype association analysis. A striking example is the CYP2D6 gene, which has greater than 70 alleles, including mutations, deletions, insertions, gene conversions and duplications (www.imm.ki.se/CYPalleles).

To have potential clinical benefit, association studies must address a ***two-fold question***: whether a phenotype is associated with genetic variations, and whether the clinical outcome distribution among genotypes is well-defined (i.e. how many sub-population groups can be predicted by genetic polymorphisms). An ideal statistical approach should have a high power to test genetic effects on the phenotypes. It should also be able to group combinations of genetic variables into clusters, where samples in each cluster share a similarly distributed phenotype. Clustered genotypes that predict phenotypes have high clinical relevance as possible diagnostic markers, which could directly facilitate future clinical decisions.

In traditional statistical theory, many multiple comparison approaches were developed.^2^,^3^ Scheffe, LSD, and Tukey’s HSD tests can evaluate the overall phenotype difference among genotype groups, but they can’t tell where the difference is. Newman-Kuels and Duncan tests are able to search for phenotype differences sequentially among genotype groups, but may result in overlapped grouping [Christensen,^2^ page 80, example 5.5.1]. Therefore, all of these approaches are capable of addressing the first part of the prescribed two-folded question: whether there is any genetic effect on the phenotype. However, none of them can provide decisive answer to the second part of the question: how genetic polymorphisms are grouped to predict the phenotype.

A restricted partition method (RPM) has been proposed^4^ to address these two aims. The algorithm ranks the genotype groups from the smallest to the largest according to the phenotype means. Then, adjacent genotype groups are merged sequentially based on a Tukey’s HSD test until it reaches a pre-specified significant level. The overall type I error is controlled by the empirical distribution constructed for the R^2^ statistic from a regression of the quantitative trait value on the final genotype grouping. This RPM method is an extension of a proposed multiple comparison approach for quantitative phenotypes. It has two important features that may affect its implementation. At first, it has inherent assumptions of equal phenotypic variance and equal sample sizes among genotype cells in Tukey’s HSD test. In practice, this assumption may or may not hold. Secondly, it uses disparate methods for genotype grouping (Tukey’s HSD test) and testing genotype/phenotype associations (R^2^). Arbitrary threshold selection for both methods may not guarantee the optimal partition.

In this paper, we propose a parametric mixture model approach to genetic association studies, where the quantitative phenotype is assumed to follow multivariate normal distribution. Differential genotype cells are allowed to have different means and variances. A sequential likelihood ratio test, i.e. one mixture vs. two mixtures, two vs. three, and so on, among subgroups defined by genetic polymorphisms indicates the significance of the genetic effect on the phenotype. The optimal partition among genotypes for phenotype prediction is determined by probability assignments from the mixture model. Therefore, this mixture model approach can simultaneously perform p-value calculation and determine the optimal genotype partition. The innovation of our mixture model includes an added penalty term to avoid nonidentifiable parameters.

The performance of our approach is evaluated with two pharmacogenetics study examples, in which CYP2D6 and CYP2B6 alleles were genotyped to predict the pharmacokinetics of a CYP2D6 substrate and CYP2B6 protein expression respectively. Because the functional relationship between CYP2D6 alleles and metabolic activity and CYP2B6 alleles and protein expression has been extensively studied, their function based partitions will serve as objective standards for assessing our mixture-model-based partitions. In addition, statistical simulations were conducted to compare performance of RPM and the mixture model.

## Methods

### Mixture model specification

The history of the mixture model’s application in genetics can be traced back as far as 1800s.^5^ Many important contributions of this approach in population genetics are well documented.^6^ We emphasize that the traditional mixture model approach has been to infer whether a phenotype from the population (such as blood pressure or drug response) is composed of multiple sub-populations determined by possible underlying, unknown genotypes. The mixture model formulation and its estimation procedure are introduced in great detail by McLachlan.^7^ We reformulate the traditional mixture model to estimate if measured genotype groups can predict a number of unknown, underlying normal mixtures in measure phenotypes.

Let us assume that we have *G* genotype groups, and every genotype group has *ng* (*g* = 1, …, *G*) phenotype samples, **y***_g_* = (*y_g_*_1_, …, *y_gng_*), where *y_gi_* is a normal random variable. We write the probability of the measured phenotype **y** as a function of the observed *G* genotype groups defining partitions of **y** and the assignment of phenotype group **y***_g_* to one of the assumed *K* clusters

(1) $$\begin{array}{l} \text{Pr}(\text{y},\text{z}|\mu ,\sigma^{2})\\ \;\;\;\;\;\;\;\;\;\;\;\;\;\;\;\;\;\;\;\;\;\;\;\;\;\;\;\;=\prod_{g=1,\ldots ,G}\prod_{k=1,\ldots ,K}{\left[\text{Pr}({\text{y}}_{g}|\mu_{k},\sigma_{k}^{2})p_{k}\right]}^{I\left\{z_{g}=k\right\}},\\ \text{Pr}({\text{y}}_{g}|\mu_{k},\sigma_{k}^{2})\\ \;\;\;\;\;\;\;\;\;\;\;\;\;\;\;\;\;\;\;\;\;\;\;\;\;\;\;\;\;={\left(2\pi \sigma_{k}^{2}\right)}^{\frac{n_{i}}{2}}\text{exp}\left[\frac{\sum_{i=1,\ldots ,n_{g}}{\left(y_{gi}-\mu_{k}\right)}^{2}}{2\sigma_{k}^{2}}\right] \end{array}$$

where *z_g_* = 1, 2, …, *K* is a multi-nominal random variable, and *I*{*z_g_* = *k*} indicates genotype group *g* follows distribution Pr(**y***_g_* | *μ_k_*, σ*_k_*). For the sake of simplicity, let’s define *s_gk_* = *I*{*z_g_* = *k*}. The log-likelihood for mixture model (1) is

(2) $$\begin{array}{l} l(\text{y},\text{z}|\mu ,\sigma^{2})\\ \;\;\;\;\;\;\;\;\;\;\;\;\;\;\;\;\;\;\;\;\;\;\;\;=\sum_{g}\sum_{k}S_{gk}\left\{\text{log}p_{k}+\text{log}\text{Pr}({\text{y}}_{g}|\mu_{k},\sigma_{k}^{2})\right\} \end{array}$$

Under the null hypothesis, the true model has only one distribution. If we fit the data by a mixture of *K*-components, any (*pk* = *p*_0_ = *p_true_*, 0 ≤ *p_k_* ≤ 1, *k* = 1, …, *K*) will achieve the maximum in (2). This problem causes not only numerical difficulties in the mixture model estimation process,^8^ but also theoretical difficulties in likelihood ratio tests.^9^,^10^ The identification problem was solved in^11^ by adding a penalty term into the log-likelihood function (2), by which the penalized likelihood function *pl*(.), (3), forces *p_k_* = 1*/K* when it reaches the maximum.

(3) $$\begin{array}{l} pl(\text{y},\text{z}|\mu ,\sigma^{2})\\ \;\;\;\;\;\;\;\;\;\;\;\;\;\;\;\;\;\;\;\;\;\;\;\;\;\;\;=\sum_{g}\sum_{k}S_{gk}\left\{\text{log}p_{k}+\text{log}\text{Pr}({\text{y}}_{g}|\mu_{k},\sigma_{k}^{2})\right\}\\ \;\;\;\;\;\;\;\;\;\;\;\;\;\;\;\;\;\;\;\;\;\;\;\;\;\;\;\;\;\;+\sum_{k}\text{log}p_{k} \end{array}$$

Our aim is the classification of the genotype cells, while Chen^11^ classify individual observations. This difference leads to distinctive estimation algorithms and asymptotic LRT.

### E-M algorithm

In the estimation step (E-step), the random variable *z_gk_* (un-observed) is estimated by (4):

(4) $$s_{\mathrm{gk}}=\frac{{\hat{p}}_{k}\text{Pr}({\text{y}}_{g}|{\hat{\mu }}_{k},{\hat{\sigma }}_{k}^{2})}{\sum_{l=1}^{K}{\hat{p}}_{l}\text{Pr}({\text{y}}_{g}|{\hat{\mu }}_{l},{\hat{\sigma }}_{1}^{2})}$$

The grouping of genotype *g* is based on the its highest probability assignment,

(5) $$group(g)=aug\;{\text{max}}_{k=1,\ldots ,K}\{s_{gk}\}$$

In the maximization step (M-step),

(6) $$\begin{array}{l} {\hat{\mu }}_{k}=\frac{\sum_{g}{\hat{s}}_{kg}y_{g}}{\sum_{g}{\hat{s}}_{kg}n_{g}},\\ {\hat{\sigma }}_{k}^{2}=\frac{\sum_{g}{\hat{s}}_{kg}\sum_{i=1,\ldots ,n_{g}}{\left(y_{gi}-{\hat{\mu }}_{k}\right)}^{2}}{\sum_{g}{\hat{s}}_{kg}n_{g}},\\ {\hat{p}}_{k}=\frac{1+\sum_{g}{\hat{s}}_{gk}}{K+G} \end{array}$$

The E- and M-steps are iteratively conducted, and the convergence is monitored based on the relative difference of the penalized likelihood function (3).

### Sequential log-likelihood ratio test

To test the number of normal distribution mixtures present in the observed genotypes, a likelihood ratio test (LRT) is conducted. The marginal penalized log-likelihood for a mixture model of *K-*component is listed in (7).

(7) $$\begin{array}{l} pl_{M}(K)=\text{log}\text{Pr}(\text{y};\hat{\mu },{\hat{\sigma }}^{2},\hat{\text{p}},K)\\ \;\;\;\;\;\;\;\;\;\;\;\;\;\;\;\;\;=\sum_{g}\text{log}\left[\sum_{k=1,\ldots ,K}{\hat{p}}_{k}\text{Pr}({\text{y}}_{g}|{\hat{\mu }}_{k},{\hat{\sigma }}_{k}^{2})\right]\\ \;\;\;\;\;\;\;\;\;\;\;\;\;\;\;\;\;\;\;\;\;+\sum_{k=1,\ldots ,K}\text{log}{\hat{p}}_{k}. \end{array}$$

The LRT is calculated by

$$\lambda =-2[pl_{M}(K_{1})-pl_{M}(K_{2})].$$

This LRT will be conducted sequentially in data analysis, i.e. (*K*_1_, *K*_2_) = (1, 2), (2, 3), (3, 4), etc., for all (*K*_1_, *K*_2_) with *K*_2_ ≤ *g*. The family-wise type I error is calculated as the cumulative p-value along the sequential test. The threshold is pre-specified at the 5% level. For each LRT step, parametric bootstrap (5,000 replications) is implemented to calculate the empirical p-value.

## Data Analysis

### Pharmacogenetic study of CYP2D6 genetic effect on tamoxifen metabolites in patients with breast cancer

**Figure 1.**
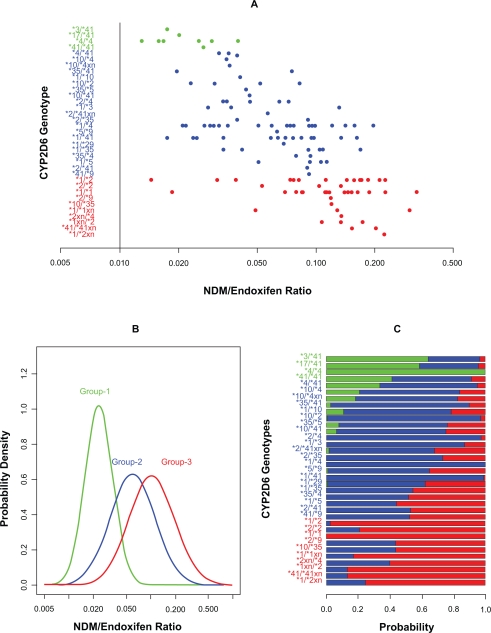
Genotype/phenotype association analysis for the Tamoxifen study. **A**) is a raw data description. The x-axis is the NDM/Endoxifen ratio in log-scale, where both NDM and Endoxifen are Tamoxifen metabolites. The y-axis denotes the 35 CYP2D6 genotypes. **B**) Thirty-five genotypes are clustered into three groups by a mixture model, which are characterized by three normal distributions. The x-axis is the NDM/Endoxifen ratio in log-scale, and y-axis is the probability density. **C**) shows genotype cell probability assignments (*s_gk_*) to each of the three predicted normal mixture components, where colored bar lengths (scaled on (0,1)) indicate the value of *s_gk_* for each mixture component. In **A**), **B**), and **C**), green, blue, and red colors represent the memberships of three clusters.

N-Desmethyltamoxifen (NDM), a major primary metabolite of tamoxifen, is hydroxylated by CYP2D6 to yield endoxifen. Due to its high antiestrogenic potency, endoxifen may play an important role in the clinical activity of tamoxifen. We conducted a prospective trial in 158 breast cancer patients taking tamoxifen to further understand the effect of CYP2D6 genotype and concomitant medications on endoxifen plasma concentrations. Thirty-five different genotypes (Fig. 1a) were determined from the 17 CYP2D6 alleles assayed. Plasma concentrations of tamoxifen and its metabolites were determined at the fourth month of tamoxifen treatment.

The NDM/Endoxifen ratio data were log-transformed for better normal mixture model fitting. However, the sample size and variance are clearly unequally distributed among 35 genotype cells (Fig. 1a). Applying the mixture model, the sequential LRTs (Table 1) suggest the 35 genotype cells were optimally partitioned into three groups. Sequential test p-values for testing mixtures (1 vs. 2), (2 vs. 3), (3 vs. 4) were 0.008, 0.032, and 0.143 respectively, with a cumulative p-value = 0.040 for the mixture model of 3 components. The genotype group with smallest log(NDM/Endoxifen) contains genotypes *3/*41, *17/*41, *4/*4, and *41/*41 (group 1 in Table 1). It has a mean of −3.76 and a SD = 0.15, and approximately 12% of the samples belong to this group. The second genotype group contains *4/*41, *10/*4, *10/*4xn, *35/*41, *1/*10, *10/*2, *35/*5, *10/*41, *2/*4, *1/*3, *2/*41xn, *2/*35, *1/*4, *5/*9, *1/*41, *1/*29, *1/*35, *35/*4, *1/*5, *2/*41, and *41/*9. Its log(NDM/Endoxifen) has a mean of −2.82 and a SD = 0.40, and 50% of the samples belong to this group. The third group contains genotypes *1/*2, *2/*2, *1/*1, *2/*9, *10/*35, *1/*1xn, *2xn/*4, *1xn/*2, *41/*41xn, and *1/*2xn. It has the largest log(NDM/Endoxifen) with a mean −2.28 and a SD = 0.42, and 38% of the samples belong to this group. Figure 1b displays the three mixture density distributions. Figure 1c shows genotype cell probability assignments (*s_gk_*) to each of the three predicted normal mixture components, where colored bar lengths (scaled on (0,1)) indicate the value of *s_gk_* for each mixture component.

RPM was conducted for the log(NDM/Endoxifen) data. Results are presented in Table 2. The RPM sequential analysis stopped at the first iteration, with p-value = 0.036. The result suggests log(NDM/Endoxifen) is significantly different among all 35 genotype cells.

### Pharmacogenetic study of CYP2B6 genetic effect on its protein expression in human liver tissues

**Figure 2.**
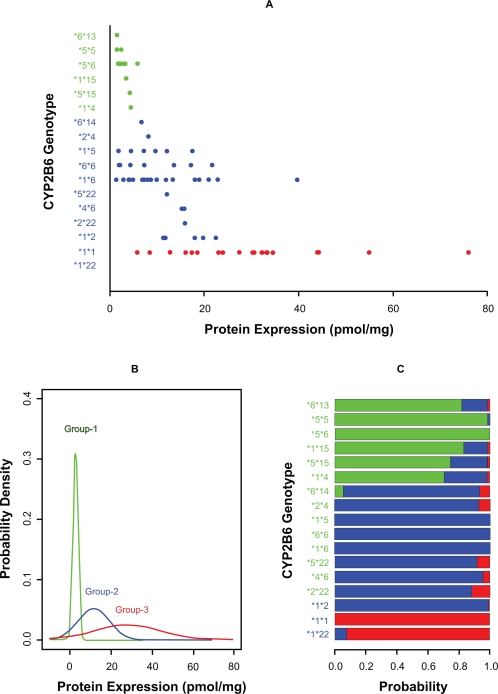
Genotype/phenotype association analysis for the CYP2B6 study. **A**) is a raw data description. The x-axis is the CYP2B6 protein expression (pmol/mg). The y-axis denotes the 17 CYP2B6 genotypes. **B**) Seventeen genotypes are clustered into three groups by a mixture model, which are characterized by three normal distributions. The x-axis is the protein expression level, and y-axis is the probability density. **C**) shows genotype cell probability assignments (*s_gk_*) to each of the three predicted normal mixture components, where colored bar lengths (scaled on (0,1)) indicate the value of *s_gk_* for each mixture component. In **A**), **B**), and **C**), green, blue, and red colors represent the memberships of three clusters.

We conducted a retrospective study, investigating the effect CYP2B6 genetic polymorphisms on CYP2B6 protein expression in 83 human liver tissues. Seventeen genotypes (Fig. 2a) were determined from 9 CYP2B6 alleles assayed (*1, *2, *4, *5, *6, *13, *14, *15, and *22). This data were recently published by our group.^12^ Protein expression level was done with western blotting in liver microsome samples. Much detail method description was described in^13^ CYP2B6 protein expression data was fitted using the normal mixture model. Sample size and variance were clearly unequally distributed among 17 genotypes (Fig. 2a). The sequential LRT (Table 1) suggests CYP2B6 protein expression levels are optimally portioned into three groups based on genotype. The sequential test p-values for testing mixtures (1 vs. 2), (2 vs. 3), (3 vs. 4) were 0.001, 0.001, and 0.153 respectively, with a cumulative p-value = 0.002 for the mixture model of 3 components. The genotype group with smallest mean protein expression contains genotypes *6/*13, *5/*5, *5/*6, *1/*15, *5/*15, and *1/*4 (group 1 in Table 1). It has a mean of 2.81(pmol/mg) and a SD = 1.64, and approximately 31% of samples belong to this group. The second genotype group contains *6/*14, *2/*4, *1/*5, *6/*6, *1/*6, *5/*22, *2/*22, *4/*6 and *1/*2. Its protein expression has a mean of 11.6(pmol/mg) and a SD = 58.1, and 52% of the samples belong to this group. The third group contains genotypes *1/*22 and *1/*1. It has the largest protein expression with mean 28.1(pmol/mg) and SD = 259.7, and 17% of the samples belong to this group. Figure 2b displays the three mixture density distributions. Figure 2c shows genotype cell probability assignments (*s_gk_*) to each of the three predicted normal mixture components.

RPM was conducted for the CYP2B6 protein expression data. Results are presented in Table 2. The RPM sequential analysis stopped at the first iteration, with p-value = 0.007. The result suggests mean protein expression is significantly different among all 17 genotypes.

## Simulation Studies

The preceding data analyses show discrepancies between the mixture model and RPM approaches. In these comparisons, RPM partitions the genotype cells into more subgroups than the mixture model. As both methods emphasize the importance of dimensionality reduction, we look favorably on the mixture model result, though both detected significant genotype/phenotype associations in their respective genotype partitions. In the following simulation studies under two epistatic models, we compare the power of the two approaches to detect genetic effects and model recovery probabilities. Of importance is the ability of both approaches to recover the true model partition.

**Figure 3.**
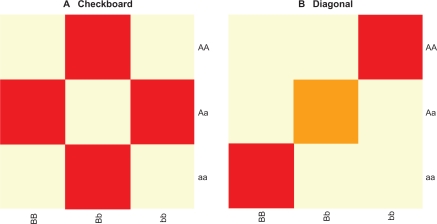
Bi-allelic epistatic models. **A**) Checkerboard model was simulated with 2 distributions among the 9 cells, with equal or unequal variances. One group consists of 4 genotype cells containing exactly one heterozygote (shaded cells), with a phenotypic mean of 0. The other five genotype cells have a higher phenotypic mean. **B**) Diagonal model was simulated with 3 distributions among the 9 cells, with equal or unequal variances. All the cells off the main diagonal have a phenotypic mean of 0. The diagonal cells (dark shaded cells) have higher phenotypic means, with the double heterozygote (light shaded cell) phenotypic mean as half that of the other two cells, but with equal variance.

Data were simulated from two 2-locus, bi-allelic models: a checkerboard model (Fig. 3a) and a diagonal model (Fig. 3b). These two models have been thoroughly described by Culverhouse.^14^ For each model, both alleles at each of the contributing loci are equally frequent (minor allele frequencies for a and b are 0.5), and the phenotype in each genotype cell is normally distributed.

Checkerboard models were simulated with 2 distributions among the 9 cells, with equal or unequal variances. One group consists of 4 genotype cells containing exactly one heterozygote (Fig. 3a, shaded cells), with a phenotypic mean of 0. The other five genotype cells have a higher phenotypic mean. Diagonal models were simulated with 3 distributions among the 9 cells, with equal or unequal variances. All the cells off the main diagonal have a phenotypic mean of 0. The diagonal cells (Fig. 3a, dark shaded cells) have higher phenotypic means, with the double heterozygote (Fig. 3a, light shaded cell) phenotypic mean as half that of the other two cells, but with equal variance.

The data were simulated as follows: assuming unrelated individuals, genotype cells are simulated independently based on allele frequencies. Given an individual genotype cell, the phenotype was generated from a normal distribution. Phenotypes were simulated under two variance assumptions. In situation 1 (equal variance), one group of cells follows N(1, 1^2^), and the other group follows N(1 + μ, 1^2^), where μ = 0.25, 0.5, and 1. In situation 2 (unequal variance), one group of cells follows N(1, 1^2^), and the other group follows N(1 + μ, 2^2^), where μ = 0.25, 0.5, and 1. 1000 datasets were simulated, each containing 500 samples. In situation 3 (Gamma Distribution), one group of cells follows a gamma distribution of mean = 1 and variance = 1, and the other group follows a gamma distribution of mean = 1 + μ, and variance = 1, where μ = 0.25, 0.5, and 1.

In both RPM and mixture model analysis, the p-value threshold is set at 0.1% level in order to make the simulation results comparable to the original PRM simulation studies.^4^ Power and model recovery probabilities from the simulations are reported in Table 3. Power was calculated by the proportion of simulated data sets where the null hypothesis was rejected. Recovery probability was estimated by the proportion of simulated data sets in which the true partition was recovered. Highlights of simulation are summarized as following:

- For models with equal variance among genotype cells (situation 1), both RPM and the mixture model methods demonstrated comparable power, but the mixture model had much higher recovery probabilities.
- For models with unequal variance among genotype cells (situation 2), the mixture model approach was more powerful and had higher recovery probabilities than RPM.
- For RPM in the unequal variance situation, both checkerboard and diagonal models had considerable discrepancies between power and recovery probability estimates. This result is due to early rejection of the RPM multiple comparison tests, making it unable to fully recover the true partition.
- Comparing the simulations under equal and unequal variance, the mixture model gained power and had increased partition recovery probability for models of unequal variance.
- If the data distribution is un-symmetric (i.e. gamma distribution), both mixture model and RPM methods have comparable performance comparing their performance in data following normal distribution, respectively.

## Discussion and Conclusion

The penalized mixture model approach for quantitative phenotypes is a novel application of the mixture model to genotype clustering in genetic association studies. As demonstrated in pharmacogenetic studies of CYP2D6 and CYP2B6, along with simulations, this mixture model method is capable of clustering local haplotypes and multi-locus genotypes to significantly reduce complexity of high-dimensional genotype space. The approach has power to detect quantitative traits loci when genetic effects on phenotypes are marginal or purely epistatic. As demonstrated in two pharmacogenetic genetic studies and simulations, it can detect both main and interactions effects of genetic polymorphisms on quantitative phenotypes.

Investigating the effect of CYP2D6 genotype on CYP2D6 metabolism of N-Desmethyltamoxifen, the mixture model approach generated three CYP2D6 genotype clusters in predicting log(NDM/Endoxifen). Before we discuss the biological rational for this classification, let us review the functionality of CYP2D6 alleles. CYP2D6*1 is the wild type allele, which codes for a fully functional enzyme. CYP2D6*2, *33 and *35 alleles contain point mutations that do not affect the catalytic properties of the protein product. CYP2D6*3–8, *11–16, *18–20, *38, *40, *42, *44 are associated with no enzymatic activity and CYP2D6*9, *10, *17, *29, *36, *37, *41 with reduced activity.^15^–^17^ The presence of multiple copies of CYP2D6 alleles (i.e. *1, *2, *35, *41) have been reported in subjects with unusually high CYP2D6 catalytic activity.^18^,^19^

Based on this prior functional information, all of the CYP2D6 alleles contained in the first genotype group in Table 1 have either no or reduced enzymatic activity. The majority of alleles in the third genotype group have either normal or high activities. There are only four heterozygous diplotypes that possess low enzymatic activity: *2/*9, *10/*35, *2xn/*4 and *41/*41xn. With the exception of *2/*35 and *1/*35, almost no genotypes in the middle group are homozygous for normal or no-enzymatic activity alleles. If these six genotype groups (*2/*9, *10/*35, *2xn/*4, *2/*35, *1/*35) were misclassified by the mixture model, they are account for only 10 out of 158 samples (6%). Therefore, the mixture model based partition is accurate according to well defined functionality of CYP2D6 alleles.

In exploring the effect of CYP2B6 genotype on expression of its protein product, 9 alleles were genotyped. CYP2B6 *1 represents fully functional expression and activity while *22 is associated with increased CYP2B6 expression.^20^ The *5 allele reduces CYP2B6 protein by about 8-fold in isolated human liver microsomes.^13^ The *6 allele has been shown to reduce function in vitro as well as the pharmacokinetics of its substrate efavirenz in clinical studies.^21^,^22^ The other alleles (*2, *13, *14, *15) have very low or completely absent function.^13^,^23^,^24^ CYP2B6*4 appears to increase^25^ or decrease (our data) activity depending on the substrate tested.

Three CYP2B6 genotype clusters generated from the mixture model reasonably reflect our expectation based on these prior studies. Genotypes in the cluster with the highest protein level are composed of only fully functional alleles, *1 and *22. Most genotypes in the lowest protein level cluster are composed of two low or non-functional alleles (*2, *4, *5, *6, *13, *15), with the exception of *1/*15 and *1/*4. Most of genotypes in the middle cluster contain one functional allele and one low or non-functional allele, apart from *4/*6, *6/*14, *2/*4, and *6/*6. If these six genotype groups (*1/*15, *1/*4, *6/*14, *2/*4, *6/*6, *4/*6) were misclassified by the mixture model, they account for 12 out of 83 samples (14.4%). In both examples, mixture model based partitions on CYP2D6 and CYP2B6 genotypes are supported by their functional information.

Comparing RPM to the mixture model approach, RPM detected genotype/phenotype associations with similar power. However, in the CYP2D6 and CYP2B6 pharmacogenetic studies, the mixture model generates three clusters for each data set, while RPM generated as many clusters as the original genotype cells. The result suggests a tendency of over clustering by the RPM method. This characteristic of RPM is confirmed in the simulation study, where RPM had a lower recovery rate for the true partition compared with the mixture model approach. Improvement in the mixture model’s recovery rate was observed when the assumption of equal variance among groups was violated, while RPM’s recovery probability was diminished.

In summary, the mixture model approach has adequate power to detect genetic effects on phenotypes and simultaneously cluster multiple genetic variables into homogeneous phenotype groups.

## Figures and Tables

**Table 1.** Mixture model based data analyses.

**Phenotypes**	**Group ID**	**Mixture Dist. and Prob. N(*μ*, σ2; p)**	**Genotype grouping**
Tamoxifen study	1	N(−3.76, 0.15; 0.12)	*3/*41, *17/*41, *4/*4, *41/*41
Log (NDM/Endoxifen)	2	N(−2.82, 0.40; 0.50)	*4/*41, *10/*4, *10/*4xn, *35/*41, *1/*10, *10/*2, *35/*5, *10/*41, *2/*4, *1/*3, *2/*41xn, *2/*35, *1/*4, *5/*9, *1/*41, *1/*29, *1/*35, *35/*4, *1/*5, *2/*41, *41/*9
	3	N(−2.28, 0.42; 0.38)	*1/*2, *2/*2, *1/*1, *2/*9 *10/*35, *1/*1xn, *2xn/*4, *1xn/*2, *41/*41xn, *1/*2xn
Efavirenz study	1	N(2.81, 1.64; 0.31)	*6/*13, *5/*5, *5/*6, *1/*15, *5/*15, *1/*4
Protein expression (pmol/mg)	2	N(11.6, 58.1; 0.52)	*6/*14, *2/*4, *1/*5, *6/*6, *1/*6, *5/*22, *4/*6, *2/*22, *1/*2
	3	N(28.1, 259.7; 0.17)	*1/*22, *1/*1

**Table 2.** RPM based data analyses.

	**Tamoxifen study**	**CYP2B6 study**
P-value	0.036	0.007
Grouping	35 groups for 35 genotypes	17 groups for 17 genotypes

**Table 3.** Simulation studies.

*μ*	**RPM**		**Mixture model**	
	**Power**	**Recovery-Probability**	**Power**	**Recovery-Probability**
Situation 1: Equal variance				
Check board model				
0.25	8%	9.7%	8.6%	49.8%
0.50	87%	51.4%	88.5%	83.2%
1.00	100%	79.3%	100%	99.8%
Diagonal model				
0.25	40%	1.3%	44.3%	33.2%
0.50	100%	44.3%	100%	61.1%
1.00	100%	86.5%	100%	97.9%
Situation 2: Unequal variance				
Checkerboard model				
0.25	0.4%	5.8%	13.5%	77.8%
0.50	16.4%	22.4%	92.9%	92.3%
1.00	99.8%	0.3%	100%	100%
Diagonal model				
0.25	0.6%	0.2%	54.4%	49.4%
0.50	15.2%	0.2%	100%	82.3%
1.00	95.4%	0.2%	100%	100%
Situation 3: Skewness (Gamma distribution)				
Check board model				
0.25	7.5%	5.5%	7.3%	49.3%
0.50	82%	46.3%	85.5%	84.2%
1.00	99%	74.3%	98.3%	93.8%
Diagonal model				
0.25	38%	2.3%	39.3%	36.7%
0.50	100%	43.4%	100%	63.2%
1.00	100%	87.4%	100%	98.9%
